# Organized Care as Antidote to Organized Violence: An Engaged Clinical Ethnography of the Los Angeles County Jail System

**DOI:** 10.1007/s11013-023-09827-3

**Published:** 2023-06-30

**Authors:** Jeremy Levenson, Shamsher Samra

**Affiliations:** 1 Department of Anthropology, UCLA, Los Angeles, USA; 2 Department of Psychiatry, Yale, New Haven, CT USA; 3 David Geffen School of Medicine, UCLA, Los Angeles, USA

**Keywords:** Clinical ethnography, Mass incarceration, Correctional medicine/psychiatry, Social movements

## Abstract

The field of medical action extends beyond the clinical encounter. Rather, clinical encounters are organized by wider regimes of governance and expertise, and broader geographies of care, abandonment and violence. Clinical encounters in penal institutions condense and render visible the fundamental situatedness of all clinical care. This article considers the complexity of clinical action in carceral institutions and their wider geographies through an examination of the crisis of mental health care in jails, an issue of significant public concern in the United States and much of the world. We present findings from our engaged, collaborative clinical ethnography, which was informed by and seeking to inform already existing collective struggles. Revisiting the concept of “pragmatic solidarity” (Farmer in Partner to the poor: a Paul Farmer reader, University of California Press, Berkeley, 2010) in an era of “carceral humanitarianism” (Gilmore in Futures of Black Radicalism, Verso, New York, 2017, see also Kilgore in Repackaging mass incarceration, Counterpunch, June 6–8, http://www.counterpunch.org/2014/06/06/repackaging-mass-incarceration/, 2014), we draw on theorists who consider prisons to be institutions of “organized violence” (Gilmore and Gilmore in: Heatherton and Camp (eds) Policing the planet: why the policing crisis led to Black lives matter, Verso, New York, 2016). We argue that clinicians may have an important role in joining struggles for “organized care” that can counter institutions of organized violence.

## Introduction

The field of medical action extends beyond the clinical encounter. Rather, clinical encounters are organized by wider regimes of governance and expertise, and broader geographies of care, abandonment and violence. Clinical encounters in carceral institutions condense and render visible the fundamental situated-ness of all clinical care. Where incarcerated people come from and what illness they come with is directly influenced by processes both external to carceral institutions and the conditions within them. And what clinicians can do for incarcerated patients, in terms of diagnosis and management, and where they can send them for additional care is also shaped by the institution in which they work and systems of which it is part. What actions clinicians take in this wider geography varies. They may seek to identify the constraints inherent to carceral institutions and promote, instead, the strengthening of public health care institutions. Alternatively, they may defend the operation and legitimacy of carceral institutions, limiting their focus to the health care provided therein.

This article considers the complexity of clinical action in carceral institutions and their wider geographies through an examination of mental health care in jails, an issue of significant public concern in the United States (U.S.) and much of the world. It uses an ethnographic focus on Los Angeles (LA) County, the site of the U.S.’s largest jail system, and the ongoing struggle over the size and scope of incarceration in the country, to understand the relationships between clinical care, institutional structures, urban governance and social movements.

Physician-anthropologist Paul Farmer offers an analytic starting point with his description of “pragmatic solidarity” (Farmer 2010:441) in settings of structural violence (Farmer et al. 2006:1686), such as prisons. In his formulation of the concept, Farmer describes a program where doctors worked with prison administrators to successfully treat Russian prisoners with tuberculosis. However, what happens when health care is used not to soften systems of punishment, but to further them?

This article answers this question in three parts. It begins with an overview of the present conjuncture in LA, where officials, until recently, had planned to solve the mental health crisis in its jail system by building a “treatment jail”. This plan presented a dilemma for jail clinicians and this paper’s authors—both working in the jail at the time—and complicates Farmer’s concept of pragmatic solidarity (Farmer 2010:441). We attempt to resolve that dilemma through a method we call “engaged clinical ethnography.” Against versions of clinical ethnography that seek primarily to improve practices *within* the clinic, this approach directs attention to the organization (or disorganization) of care produced by institutional and extra-institutional political forces, within and outside the clinic. It is *engaged* as it is directly informed by and seeking to inform already existing political struggles happening outside the clinic.

The second part presents fives themes from our collaborative ethnography that demonstrate tensions in jail care. It highlights how the jail’s limited institutional capacities frustrated attempts at providing high-quality clinical care, and how these constraints helped support the plan for a “treatment jail”. We also show how these constraints led some clinicians, including Author 2, to join efforts outside the institution to contest the legitimacy of the jail plan.

In the final section, we put Farmer’s pragmatic solidarity (Farmer 2010:441) in conversation with Ruth Gilmore and Craig Gilmore’s framework that takes carceral institutions to be institutions of “organized violence” (Gilmore and Gilmore 2016). This framework addresses a gap in Farmer’s formulation: his under-theorization of the state. Gilmore and Gilmore’s framework for the state—as a set of ideological and institutional capacities determined by struggle—helps connect jail clinical work to its wider political context. To complement this framework of organized violence (Gilmore and Gilmore 2016), we introduce the term “organized care.” The term describes an alternate vision for what the state could provide, both in ideological meaning and institutional practice, which has inspired ongoing social movements in L.A.

We conclude by suggesting that a framework of organized violence (Gilmore and Gilmore 2016) and organized care demonstrates the need to broaden the field of action beyond the clinical encounter, whether that encounter occurs in a hospital, jail or detention center. In joining already existing struggles, clinicians, we argue, may practice a solidarity that is both pragmatic and liberatory (Dubal, Samra, and Janeway 2021).

## Background: Mission (Im)Possible

In 2018, the year we began working in the jails, LA County announced a new campaign, “Mission Possible.” The initiative sought to “attract idealistic medical professionals” to the cause of a “mission-driven transformation” of LA’s jail health care system (LA Chief Executive Office 2018). The county hoped to address what they described as a shortage of clinicians capable of carrying out its new vision of “social justice” jail medicine. The campaign’s name intimated the enormity of the problem. During the past four decades, the LA County jail system has been a central node in California’s massive carceral archipelago and become a national symbol of the violence at the center of the U.S.’ carceral state.

The LA County jail system is operated by the county’s Sheriff’s Department (hereafter ‘LASD’). Like jails across the U.S., it receives people arrested by police officers and Sheriff Deputies, detains them as they are processed by courts and then, if they are convicted, coordinates their transfer to state prisons. Most detainees are held for a short time and the majority are held pre-trial. The churn typical of jails has been made more chaotic, however, by changes in California’s wider systems of punishment and health care—affecting both the volume and composition of people in custody.

California was a forerunner in the U.S’ turn to incarceration in the 1980s and 1990s (Gilmore 2007). By the early 2000s, the state increased criminal legal spending by over $600 billion, from its 1982 level, building over 23 prisons between 1980 and 2010 (Zimring and Hawkins 1994). Indigenous, Black and Latinx people were disproportionately affected. A range of actors—elected state and county officials (Gottschalk 2015), judges, Corrections Officers’ and police unions (Page 2013), news media and property owners (Gilmore 2007)—spurred this transformation.

In 2011, the Supreme Court’s *Brown vs. Plata* decision, which ruled that overcrowding in California’s prisons amounted to “cruel and unusual” punishment, appeared to promise an end to the relentless growth of incarceration (Simon 2014). Since then, only one prison has been constructed, a prison health care facility in 2013. However, California also redistributed prisoners to counties by increasing the number of short sentences to be served in county jails and reclassifying felonies as misdemeanors. These changes shifted both people and custodial responsibility from state to county (Reiter and Pifer 2015).

Over this period, California also underwent a transformation in its mental health care system. In the 1970s and 1980s, state officials failed to finance the community mental health system originally promised after the closure of many state mental hospitals. By the 1990s, an orientation towards cost savings via managed care, reducing public services and “community care” became common sense to policymakers, who looked to non-profits and the private sector to provide care (Braslow et al. 2021). So, even as officials embraced the public sector’s role in imprisonment, California, like states across the country, largely withdrew from the provision of long-term mental health care. This institutional retrenchment occurred in tandem with a worsening crisis in housing, a decline in public benefits, the loss of jobs and the growth of the criminal legal system (Ben-Moshe 2017). Together, these changes contributed to a rise in the prevalence of mental illness among incarcerated people in prisons and especially jails (see Torrey et al. 2010 and Bronson and Berzofsky 2017 for national data).

In recent decades, the situation has worsened. Between 1995 and 2014, California’s acute psychiatric inpatient beds declined from 29.5 to 17 per 100,000 (California Health Care Almanac 2018). LA County alone faces a deficit of over 5,000 psychiatric beds (California Hospital Association 2018). A 2019 study commissioned by LA’s elected leaders confirmed a significant shortage in mental health services at every stage of care (Sherin 2019). Meanwhile, from 2009 to 2019, the prevalence of active mental health cases amongst inmates in California jails rose by over 40% (Franco 2020).

In LA, prior to the pandemic, jails averaged 17,000 inmates at a time and over 120,000 bookings per year (LASD Custody Report 2019). While a decline from its peak of 22,000 in the 1980s, it remained over 5000 higher than the facilities’ stated capacity (Board of State and Community Corrections 2018). Throughout this era, poor, Black and Latinx male and increasingly female residents of LA have been disproportionately represented in the jail (LASD 2019). The longstanding structural race, gender and class violence has also increasingly affected people with disabilities and chronic medical and psychiatric illness: the rates of mental illness grew from 14% to nearly 30% between 2009 and 2019 and estimates suggest up to 65% of jail inmates at any time need substance use disorder services (LASD 2019).

The jail health crisis has been deepened by the LASD’s malfeasance. The LASD has been the subject of repeated high-profile class-action lawsuits and governmental investigations since the 1980s, for abuses such as corruption, use of force, jail overcrowding, racist deputy gangs and the abuse and neglect of people with mental illness and disabilities. Since 1997, the Department of Justice has engaged in almost continuous investigation of LASD’s jail mental health care.

In response, the LASD engaged in multiple efforts at health care system reform and expansion in the late 1990s and early 2000s. It created designated and licensed medical and psychiatric spaces within the jail, expanded its custodial staff and doubled its mental health staff (Lara-Millan 2021). The LASD also worked with County officials to re-allocate over $20 million dollars in mental health funds towards its own use, despite opposition from LA’s Association of Community Mental Health Agencies, per Armando Lara-Millan’s archival research. As he argues, the “medicalization” of the jail paid clear financial dividends in an era of austerity and declining legitimacy of incarceration. With pressure from the Department of Justice continuing into the late 2000s, the County and the LASD proposed what appeared a common-sensical and benevolent solution: use state funds to replace its oldest jail with a new “correctional treatment facility,” continuing the logic of expansion by blurring the boundary between incarceration and treatment.

Community opposition to sheriff and jail violence, however, forced LA’s leaders to reconsider the plan and consider alternatives to incarceration. The County, in response, established an “Office of Diversion and Re-Entry” and hired a health consulting agency to revisit the jail plan. The consultants confirmed that a new jail was necessary as they expected medical and psychiatric illnesses in jail to increase indefinitely as the jail population increased (Health Management Associates 2015). In other words, they legitimized the expectation that people with serious mental illnesses and chronic illnesses would increasingly churn through the jails. The county’s “Mission Possible” campaign complemented this expectation; social justice-driven jail clinicians were needed to care for the growing population of sick inmates. If, however, this expectation was rejected and the expected increase in the jail’s population was itself considered a health crisis, what alternative role could clinicians and health systems play?

During our time working inside the jails, a coalition that offered an alternative script led LA’s officials to doubt their plan. The JusticeLA coalition, composed of several community organizations, mobilized experts and advocates to protest the incarceration of people with substance use and psychotic disorders, and pushed instead for “care, not cages.” Organizers from this coalition sought out both Author 1 and Author 2, who joined their effort.

## Setting the Scene: Beyond the Jail Clinic

In spring 2019, Author 1 and Author 2 arrived at a meeting with Deputies from the offices of the Los Angeles County Board of Supervisors. The meeting had been arranged by community organizers. It was one of several meetings between the County’s elected leaders and a group of physicians, which included emergency medicine doctors and psychiatrists. The topic was the nearly $2-billion proposal to build a treatment-oriented jail.

The physicians explained why they had joined the JusticeLA coalition of activists protesting the proposal. One emergency medicine physician noted: “From a clinical perspective, the need for additional beds isn’t unique to the jail,” he said. “Every day, patients come into the public ED unable to find mental health beds, addiction treatment, primary care doctors. There are so many other things we need.”

Author 2 joined: “The thing about the jail setting is that, despite our efforts, it is just not oriented towards care. And it is not up to community standard. For, say, substance use treatment, it is behind by decades.”

A psychiatrist was still more emphatic: “What we see in the jail is 100% the failure of our community mental health system. A new jail is only more of the same…and gets us nowhere.”

JusticeLA’s arguments proved persuasive (Clayton-Johnson, Samra, and Levenson 2021). In August 2019, the Board of Supervisors canceled its contract for jail construction. They also commissioned a working group to pursue alternatives to incarceration, which published a blueprint, “Care First, Jail Last,” for a plan based in decentralized systems of care (LA County Alternatives to Incarceration Work Group 2020). The question that loomed was: how could a new system be borne from the old not only practically but also politically, with the support of clinicians and health workers?

## Methodology: Engaged Clinical Ethnography

Over a year before this meeting, we both began working in the LA jail system, in the setting of the Mission Possible campaign. Author 1, an anthropology doctoral student and medical student, had been granted access to conduct an ethnography that sought to explore how the jail setting affects the meaning and practice of mental health care. He conducted fieldwork from 2018 to 2020, shadowing over 20 clinicians across multiple settings within the jail and interviewing over 25 clinicians. Between the fall of 2018 and the summer of 2019, he conducted approximately 10 h per week of fieldwork and between summer of 2019 and March 2020, he conducted between 10 and 30 h a week of fieldwork. Clinicians included nurses, psychiatric technicians, social workers, psychologists, psychiatrists, pharmacists, medical doctors and incarcerated peer caregivers. He integrated this research with participation observation outside the jail, following forensic psychiatrists into courtrooms and observing public meetings and hearings on jail mental health, during which he took detailed notes. In total, Author 1 observed 12 such public sessions in person and reviewed recordings of 10 additional sessions. His ethnographic research was approved by his institution’s Committee on Human Research.

Author 2, an emergency medicine physician, had been recruited by the county to a clinical and administrative role in the jail. For more than 4 years, including the entirety of Author 1’s fieldwork, Author 2 worked clinically in the jail’s urgent care clinic. He also had an administrative role, helping manage the health service’s efforts to coordinate transitions of care for patients upon their release.

We met just as we were starting our roles in the jail. We had been invited to a meeting led by community organizer Mark-Anthony Clayton-Johnson, who was building a network of health workers to oppose the county’s jail plan. The questions Clayton-Johnson raised—of what plan the county might pursue instead of a new jail, and how to build a coalition to persuade the County to abandon its plans for a new jail—widened our imagined field of ethnography and clinical action, respectively. It also spurred our collaboration.

What began as Author 1 observing Author 2 in the clinic evolved into a collaborative method we call “engaged clinical ethnography.” Clayton-Johnson’s project drew our attention to not just the institutional constraints on care in jail but also the political subjectivities and imaginations of clinicians under these constraints. This provocation shifted our focus from the clinician and patient to the clinician and institution. In scheduled conversations, we put the themes and findings from Author 1’s observations and interviews in dialogue with Author 2’s reflections, as a clinician working in the jail and a participant in closed-door meetings with leadership. While our collaboration privileges the perspective of Author 2, an interlocutor in Author 1’s study, it also elicited ethnographic data that would have otherwise been inaccessible to Author 1.

Our different positions, as ethnographer and clinician/administrator, generated a productive conversation about the limits to our respective knowledges. Joel Harvey, a prison ethnographer and later prison psychologist, has described the specific affordances each role offered him in the context of prison (Harvey 2015). We had a similar experience: where Author 2 had unique insights from caring for patients and specific knowledge about the health system’s administration, as an ethnographer, Author 1 had, unexpectedly, access to more spaces across the jail complex. In a different way, given Author 2’s role high on the clinical hierarchy and Author 1’s outsider observer status, our vantage enabled us to develop a broader view of the health system’s organization. At the same time, these positions shaped our interactions with jail clinicians: those lower on the hierarchy than Author 2 may not have been as forthright and also more suspicious of Author 1, since they were already frequently surveilled as part of jail lawsuits.

Our “engaged” approach provoked us to consider how what was happening outside the jail directly shaped the conditions inside it. Author 1 joined Author 2 as he participated in actions outside of the jail, such as meetings with organizers, activists and elected officials and public gatherings, including hearings by the County Board of Supervisors and planning sessions of County working groups in 2018 and 2019. From there, we could compare the political “common sense” of policymakers and advocates to that of clinicians working within the jail.

This synergistic ethnographic account, which emerged from our iteratively developed method, follows the longstanding methodological tradition of collaborative ethnography, wherein researchers and their interlocutors co-produce ethnographic texts and its attendant knowledges (Lassiter 2005). Collaboratively, we draw on and extend the methodologies from clinical ethnography, ethnographies of prisons and ethnographic engagements with social movements.

Traditional clinical ethnography typically foregrounds the complex relationships, discourses and practices that are enacted in clinical interactions between providers and patients, occurring most often but not exclusively in the clinic. Recent major ethnographies exemplifying this focus include those by Garcia (2010) and Sufrin (2017). Anthropologists have argued that an ethnographic approach may illuminate routine forms of mystification (Taussig 1980) and dehumanization (Kleinman and Kleinman 1991) that occur in clinical interactions. However, while many such studies point to structural forces shaping clinical phenomena, how such structures are being formed outside the clinic may fade to the background (Scheper-Hughes 1990). For this reason, others have broadened their focus to include health providers and workers and how they interact with the extra-clinical systems and structures (Wendland 2010).

These extra-clinical approaches have also sometimes drawn anthropologists directly into political struggle. Adrienne Pine, for example, joined nurses and other healthcare workers in the Honduran Resistance movement. Her scholarship joins traditions of “engaged” anthropology, which declares its own political commitments at the outset. While Sherry Ortner has recently noted an “engaged turn” in anthropology (Ortner 2019), these traditions date at least back to writings collected by Harrison (1991) and Hale (2008). Here, we follow scholars like Claire Wendland and Pine who connect the subjectivities of health workers in the clinic to wider sociopolitical struggles, considering their scholarship examples of “engaged clinical ethnography”, whether or not they formally described it as such.

Prison ethnography faces a different problem than clinical ethnography’s focus on the micro case study: the structure of institutional power fundamentally limits the possibility of participant observation. Prisons do not just tightly regulate space and relationships; they also control the flow of knowledge, dictating who can enter and what information and settings are observed. It was partially for this reason that, after the prison rebellions of the 1970s, there was a decline of detailed, immersive prison ethnographies^1^ in the U.S. (Rhodes 2001; Wacquant 2002). This decline has meant that some transformations, such as the rise of correctional health care, have been less documented ethnographically.

Nevertheless, there have been major contributions to the field of prison ethnography from anthropologists working outside the U.S, especially in Central and South America, Canada and Europe, where research access has less frequently been an obstacle (see Drake, Earle, Sloan, 2015 and Sozzo 2022). Particularly relevant for us was research that investigated shorter stay institutions (Fassin 2017) and those that explored the braiding of life outside institutions with life within them (Cunha 2008). In this literature, correctional health care, clinical staff and treatment programs have remained mostly in the background. James Waldram’s ethnography of a prison treatment program for sexual offenders in Canada, however, represents an important exception (Waldram 2012). While he focuses on the experience of inmates and not on clinicians, Waldram persuasively highlights the tensions inherent to clinical work in prisons and “therapeutic intervention” more generally, wherein the subjects and objects of treatment and care are often in conflict.

In recent years the clinic has been an unexpected site for physician-anthropologists to re-enter correctional institutions in the U.S. Kimberly Sue follows women who use drugs into and out of prisons, identifying what she calls the “carceral-therapeutic state” (Sue 2019). Carolyn Sufrin also focuses on the trajectories of criminalized patients but focuses on a single jail and the meaningful, if ambivalent, form of care practiced therein (Sufrin 2017).

However, anthropologists have pointed to the institutional and ideological constraints that can delimit ethnographic research within carceral institutions (Feldman 1991), even when access is granted. Neither the outsider researcher nor the insider employee can truly “participate” in the experience of incarceration (see Walker 2016 for an exception). Such constraints make qualitative work vulnerable to normalizing disciplinary power; for this reason, Lorna Rhodes has argued that “the most pressing need for the study of prisons is to challenge the terms of the discourse that frames and supports them” (Rhodes 2001:75).

Responding to these challenges, some, like Rhodes in her ethnography of a maximum-security prison, foreground institutional discourses such as “dangerousness, self-control and choice” (Rhodes 2004:12). Others have reconsidered the boundaries of “the field” of the prison. Orisanmi Burton, for example, has turned towards letter writing with imprisoned intellectuals for ethnographic insight into institutional tactics of counterinsurgency (Burton 2021). Judah Schept also studies the prison from its outside, examining the discourses, logics and “carceral habitus” that structure their social reproduction (Schept 2015).

Rather than accept the prison as a natural part of a social landscape, these ethnographers foreground the political struggles occurring within and outside of carceral institutions. In doing so, these scholars add to anthropological scholarship on social movements. While some anthropologists have explored the messy, conflict-ridden experiences of activist groups and political formations engaged in formal protest (see Juris 2008 and Graeber 2009 for studies of transnational movements), others have taken more indirect and/or less traditional approaches, broadening the scope of what is considered resistance (Gregory 1998) and attending to the ideas that animate struggles. Burton and Schept model the latter: Burton focuses attention on the radical vision of revolutionary prisoners (Burton 2016, 2021), and Schept explains the persuasion of carceral ideology (Schept 2015). Both are engaged in so far as they contribute to struggles against the idea of prisons as inevitable (Gilmore 2008).

Our method synthesizes these three ethnographic traditions. We draw on our access within the jail to gain more knowledge about the terms, discourses and contradictions sustaining the carceral state. However, we pivot from the traditional focus of clinical ethnography to foreground the context for clinical care and the political subjectivities, and non-clinical actions, of clinicians. Pine has argued that the social-change oriented ethnographer is “akin to that of a movement or union organizer” (Pine 2013:143). Here, like Pine and other engaged clinical ethnographers, we seek to better identify what it may take for more jail clinicians to take action beyond the clinic and join the struggles into which Clayton-Johnson invited us—which is not always straightforward.

## Disorganizing Care

Arrests and incarcerations disorganize lives—lives that are frequently already in crisis. Rent is not paid, jobs may be lost, social lives are put into disarray and health care can be interrupted. The chaos of jails has been documented ethnographically, such as in John Irwin’s account in California (1985) and Issa Kohler-Hausmann’s study in New York City (2018). The role health care plays in this chaos, however, is variable.

On starting our respective positions in the jails in 2018, we encountered a situation far different from that advertised in the “Mission Possible” campaign. Here, we present themes from the ethnographic data that emerged from Author 1’s observations of the jail clinical environment and reflections from Author 2’s participation in health system leadership. Together, this data reveals the confrontation we observed between the promise of “mission-driven transformation” and the reality of a health bureaucracy organized around controlling cost and liability and in partnership with custodial authorities.

### Alienated Care

Many clinicians Author 1 spoke to described how the opportunity to serve marginalized patients and practice “social-justice medicine” had inspired them to work in the jails. However, what they discovered was a clinical environment of staff who rarely shared their sense of purpose and one made more difficult by institutional and administrative constraints. One physician working in the medical intake area summarized to Author 1 her impression of the clinical environment.

Fieldnote excerpt:

‘I like to take my time, as you probably noticed,’ Dr. Ralph laughs. We are on lunch break after a morning of seeing patients in the clinic adjacent to the jail’s intake. Roughly 400 people a day are processed there. Dr. Ralph had seen five patients that morning, spending at least thirty minutes with each.

‘They [the nurses and officers] get a bit frustrated with me,’ she smiled. ‘They are under pressure to move the patients into housing as fast as possible. So they prefer people like Dr. Allen. I call what he does drive-by medicine. You’ll see what I mean.’ A few weeks later, I did: barely turning to look at patients, Dr. Allen asked a rapid-fire series of leading questions. The encounters lasted a couple minutes. A glance at a patient’s medical record, and past incarcerations, seemed to tell him everything he needed to know.

‘Dr. Allen has been here forever. Quick. Efficient. He’s appreciated.’ Dr. Ralph adds that Dr. Allen shares a sentiment popular in the clinic: most patients are just looking for an extra mattress, wheelchair, or other supplies to make their incarceration more comfortable.

‘I think it is important to take my time, though,’ Dr. Ralph tells me. ‘It may be the only time many of these patients see a doctor outside of an ER. There are eye rolls but nothing more.’ (2019)

Over time, Author 1 noticed other staff appear irritated as Dr. Ralph would pause to teach or continue a conversation about a patient. Many other clinicians who Author 1 approached declined participating in Author 1’s study; some asked whether, instead of observing, Author 1 could instead see patients on his own. Driven by the institutional focus on mitigating death, efficiency, not care, was their priority.

In the mental health intake area, Author 1 observed staff to be similarly overwhelmed by volume and the demands put on them by the institution. The top concern was assessing the patient’s risk for self-harm, given the high rates of self-harm and suicide in jail settings, and the regulations introduced by class action lawsuits. Many clinicians noted how the constraints of the setting made psychiatric interviewing very difficult.

As Author 1 learned, after booking, inmates with suspected psychiatric illness are taken to an observation area. There, they are handcuffed to the ground and given only a “safety gown,” a smock made of hard-to-tear nylon. If they continue to appear psychotic or state that they are suicidal, they are taken to an extended psychiatric triage area, or the “EPT,” which can hold up to 90 inmates. It was almost always fully occupied. One EPT psychiatrist described to Author 1 how he manages the challenges of the setting.

Fieldnote excerpt:

‘It’s basically a community psych emergency room,’ says Dr. Smith, ‘except here they are caged like in a zoo and act like it.’

A specialist in emergency psychiatry, Dr. Smith splits his time between a county hospital and the jail, where he has been working for nearly a decade. He mostly likes the challenge and feels a sense of responsibility. ‘These are some of the sickest patients that I see,’ he says.

However, he also finds the setting exhausting. At the beginning of shifts, he cleans his table and keyboard with an antimicrobial wipe. The act appears to give him a modicum of control over a place where he has little.

A corrections officer and social worker assign him the ten patients he will see every shift. It usually depends on which patients are most disruptive. Today we hear echoes of one patient screaming repeatedly and another banging against their cell door. Later he says with exasperation: ‘Some days it is so loud in here, I can’t even hear myself think!’

I ask what differences he has noticed between the county and the jail.

‘Well, in both places, medical care is my primary job. If someone is going to die, it is from a medical cause – withdrawal, delirium or some other medical cause of altered mental status. That or suicide. But here, it is harder.’ He explains: ‘In an ER, there are nurses who know the patients, collect labs and will tell you if something needs attention. But here, they only really have staff to hand out the meds. Otherwise, it’s just me, a couple social workers, the Deputies and then up to 90 patients. And I don’t have labs. So, I need to just focus on whether they are safe right now.’

‘Adding to all that, there are very rarely doctor-to-doctor referrals.’ He explains that he almost never hears from the person who decided which patients needed to be sent to the EPT. Most days, he barely interacts with any other mental health staff, except the social worker. One day I ask the last time he was in a room with the full jail mental health service. He couldn’t remember. (2019)

Both Dr. Ralph and Dr. Smith stated that they found jail health care meaningful enough to continue working, despite the setting’s constraints, which include the condition of their patients, the pressure they received from peers, administrators, and custodial staff to process and house patients, and the disorder of the health service. They focused on the patient in front of them, rather than the system of which they were a part. Others, however, found these constraints too significant and quit during the time Author 1 was conducting fieldwork.

Dr. Ralph and Dr. Smith’s reflections draw attention to a question that frequently came up in jail clinical work: what is the appropriate standard of care in a jail setting?

### The Jail Standard

Jail health care is regulated differently from the rest of the health care system, where federal reimbursements are contingent on following national standards. While jail health care standards exist, to what extent carceral institutions follow them depends on the operators of those institutions, the power afforded to state and county oversight agencies and the consequences of class-action lawsuits. Counties thus have significant autonomy in determining the quantity and quality of care they provide (Dolovich 2022).

In the LA jail system, clinicians would frequently identify discrepancies between the jail standard of care and the standard of care they used in the community. Unlike prisons, large jails in urban settings often employ clinicians who also work in non-jail clinical settings. Dr. Smith and Dr. Ralph represent two such examples. For these clinicians, the discrepancies in the diagnostic, therapeutic and preventative modalities available to them were unmistakable.

One object of recurrent scrutiny was the jail’s minimal substance use treatment. While other counties such as New York City have provided a range of Medication-Assisted Treatment^2^ (MAT) services for decades, in 2018, LA’s MAT services were limited to pregnant inmates. The absence of treatment was especially glaring since the jail’s own health data suggested that up to 65% of its inmates had a substance use disorder (LASD 2019). As a result, people incarcerated for drug charges would not be offered treatment while in jail and people on MAT in the community would go through forced withdrawal during their 1st weeks of incarceration.

Author 1 shadowed the clinician who managed the jail’s alcohol detoxification program. She explained how leadership told her that they need to offer alcohol detoxification, rather than opioid detox, because alcohol detoxification is life-threatening.^3^ Part of her job was to review patient charts, separating those using alcohol from those who used other substances, such as opioids. She relayed how clinical frustration seeped into charts. She paraphrased one physician’s note that included the following: “Per patient, ‘why the hell was I arrested for drug use if I was not going to be offered treatment?’ Author agreed with patient.” Another physician tried surreptitiously prescribing Buprenorphine to a patient against policy, only to be disciplined by her supervisors.

The topic of MAT frequently arose in administrative meetings. Author 2 recalled his surprise when some administrators demonstrated low clinical understanding about MAT services. He also heard from his supervisors directly that they just did not see substance use treatment as a priority and observed their frustration mount when staff would continuously bring it up. They considered MAT administratively and financially difficult and prone to abuse by patients. It also became evident to Author 2 that they just did not believe there was anything wrong with the forced withdrawal and abstinence that incarceration induced. It was not a surprise, then, that we observed a revolving door of clinicians at the jail hired to lead the jail’s MAT program. Over our 3 years, we observed four such clinicians quit.

The availability of appropriate housing for people identified as psychiatrically ill was also a point of contention both externally, in the recurrent lawsuits and media stories described above, and internally. The sheer volume of psychiatrically ill inmates make the LA jails the largest provider of institutional mental health care in the U.S., a statistic frequently noted in public and policy debates.^4^ However, jails are fundamentally not mental health institutions. Jails are organized for punishment and only reluctantly accommodate mental health services, which are created primarily in response to court injunction. Author 1’s observations in the jail testified to this banal but important difference.

While the LA jails had at least 1500 people diagnosed with severe mental illness, they had 32 beds that met formal inpatient criteria. During our time in the jails, the waiting list for these beds ranged from forty to nearly two hundred people. Most mental health clinicians Author 1 spoke to suggested that this list far underestimated the need; since everyone knew the shortage, the hurdles to be added to the list were significant. When Author 1 asked Dr. Smith how many patients in the jail’s EPT needed inpatient psychiatric hospitalization, he replied: “That is THE question! I would say most! These are some of the sickest patients I see.” While not all clinicians shared the same projection, most agreed that there were at least several hundred needing inpatient hospitalization. Furthermore, there was unanimous sentiment, amongst those with whom Author 1 spoke, that the jail system lacked the capacity needed to meaningfully care for patients in need of psychiatric hospitalization.

Finally, some clinicians expressed concern about the limitations on their tools of disease prevention. Dr. Ralph was among several who noted the absence of influenza vaccines. She was especially frustrated given the increased rates of HIV-AIDS among incarcerated people, particularly those in jail settings, as well as the inherent risks of respiratory transmission in settings as dense as the LA jail system.

These concerns were exacerbated with the arrival of Covid-19. In March 2020, the jail’s leadership sent out an e-mail to staff with the new testing and quarantine criteria. The announcement did not stipulate that standards in the jail were different from those in the community. But Author 2, and many of his colleagues, recognized the difference. When he raised the issue, he was told they had no other option, due to the limits on their capacity to quarantine individuals under investigation. Within the next 6 months, after at least seven Covid-related deaths, the LA jails’ Covid plan was subject to a class-action lawsuit.^5^

The myriad concerns raised by jail clinicians inspired both conversation and proposals for change—change that was subsequently met with resistance from health leadership.

### Disorganizing Workers

Many clinicians described the resistance they encountered in advocating for improvements in the health system. Those involved in the MAT efforts, for example, explained that the leadership was simply not interested. Others, brought in to help the jail become a prevention-focused “medical home” model of care, were disappointed as they saw leaders turn instead towards urgent care. Dr. Ralph offered Author 1 a metaphor:

Fieldnote Excerpt:

‘At the top of the mountain, there are leaders who keep things in order. Physicians who are part-time and just see patients are at the bottom and mostly do as they please. Full-time physicians who express interest are promoted. If they are efficient, do as they are told and keep the system intact, they are kept there. But if they cause problems, they are pushed off the mountain [leave the jail].’ (2019)

To Dr. Ralph, the health system was organized, in other words, just like the Sheriff Department—in a vertical structure of authority. As a result, she resigned herself to focusing on immediate patient care.

At first, Author 2, hired to support the implementation of a community health worker-driven program, assumed expanding the system’s capacity was part of the job. So when, shortly after being hired, he was approached by co-workers who wanted to brainstorm ways to improve the jail’s transitions of care, he readily joined. Together, the group discussed how to install primary care referrals, strengthen linkages to community substance use treatment and better coordinate specialty services. Their group was organized independently of the jail’s health service and operated outside of its hierarchy and bureaucracy. It consisted of health staff, community health workers and social workers.

When his supervisors learned about this group, they discouraged Author 2’s involvement and believed the effort was ill-construed. One supervisor pointed out how individuals with diabetes had better control of their blood sugar in custody, which he considered an example of why it is futile to coordinate post-release care. In other words, low medication adherence and fragmented care for this population was considered inevitable. This example was one of many that supervisors used to argue that such groups were not worthwhile or central to the purpose of the jail health service. Leadership eventually dissolved the group.

Finding a health system hostile to change, many new hires quit nearly as soon they arrived. The high labor turnover left the system administrators, and the County, where they started when the Mission Possible campaign was launched, with a shortage of clinical staff. In response, the County pivoted to hiring private-equity backed staffing agencies to recruit and manage non-unionized, contracted care providers.

### Carceral Common Sense

In 2019, opposition to LA’s plan to expand its jail system grew to a fever pitch. However, some jail clinicians who participated in public forums expressed tentative support for the plan. During our time in the jails, the County’s plan often came up. While some were indifferent, many were in favor of the plan, noting that it could involve more positions and more work for health care staff, as well as improved facilities. They shared the County’s assessment that some of the jail facilities needed to be replaced.

Several mental health clinicians also expressed their support for the rationale of the plan. To them, the promise of additional inpatient beds, and mental health housing areas in general, was persuasive. They noted that their patients with mental illnesses were housed in settings originally designed as punitive segregation areas and officers, not nurses and psychiatrists, were their first responders. Clinicians observed how patients would most often worsen, leading to high rates of self-directed violence and suicide. As a result, clinicians like Dr. Smith viewed the plan as commonsensical. He and others acknowledged that many inmates needed long-term psychiatric services, not incarceration. However, in the absence of plans to fund those services, improving jail mental health with expanded facilities was a practical compromise.

### Organizing for Care

The support for the jail plan demonstrates how the contradictions within and the discourses emerging out of the jail can lead to an alignment with the institution. However, not all clinicians believed new infrastructure alone could resolve the problems they observed within the jail. Author 2 represents one example of the latter, as it was the crisis within the jail that inspired him to take action outside of it.

What struck Author 2 was the expectation implicit to the jail plan that a new facility would resolve many of the tensions he observed in practice. However, especially from the urgent care setting where he practiced, he witnessed many issues that were direct outcomes of the incarceration setting, such as injuries, patients’ refusing to talk for fear of retaliation as well as officers not bringing patients in a timely fashion and influencing clinical assessments. Moreover, his participation in meetings with leadership revealed the degree to which the notion that incarcerated patients were difficult and less deserving of care undermined outward commitments to social justice medicine. Given the health system’s reluctance to increase its technical capacity, Author 2 had little hope that a new facility would resolve these political and regulatory problems.

Due to his wariness about the hopes the County placed in the jail plan, Author 2 decided to join meetings with the wider JusticeLA coalition. There, he discovered their focus on supporting efforts to improve transitions of care for incarcerated people, as well as the availability of community-based substance use treatment and acute inpatient mental health beds. Along with a few other jail clinicians, he joined the coalition and publicly expressed his opposition to the jail plan. Within the jail, one of his supervisors condemned him. In addition, his administrative duties were reduced and he was assigned the least desirable shifts.

The defeat of the jail plan proved, in some ways, an inflection point. Funding for MAT, which had been elusive, emerged and the jail finally began offering Buprenorphine to a limited number of inmates. In addition, more financial and administrative support was given to diversion and re-entry work, helping secure the release of thousands of people with psychiatric illness. Author 2 also observed many non-jail-based colleagues become engaged in jail-related work.

Thus, it was struggle outside the jail that introduced new opportunities for clinicians and health workers within the jail and throughout the county. This experience encouraged Author 2 to revisit and explore alternatives to Farmer’s pragmatic solidarity (Farmer 2010:441), which had first inspired his work in the jail.

## Pragmatic Solidarity and Carceral Humanitarianism

In Farmer’s elaboration of pragmatic solidarity (Farmer 2010:441), his emphasis on the *pragmatic* is a call to action. Farmer advocates moving from simply recognizing and expressing outrage at social injustice to the work of delivering services, designing and implementing programs and saving lives. That such action is *rapid* highlights the temporality of the actions he calls for: he wants us to find “short-term strategies to move vital goods quickly from settings where they abound…to places where their utter absence exacts a daily toll of suffering and death” (Farmer 2010:550).

In describing his and his colleagues’ work to develop a treatment program for Russian prisoners with multi-drug resistant tuberculosis, Farmer demonstrates pragmatic solidarity (Farmer 2010:441). By presenting themselves as “TB specialists” rather than social scientists or human rights investigators, Farmer’s team was invited into the jail for their clinical expertise. Doing so enabled them to “make common cause with the destitute sick” (Farmer 2010:440), which he considers a more meaningful form of solidarity than strictly legalistic or policy approaches to human rights violations.

The U.S. carceral state is exemplary of the structural violence against which Farmer advocates that health workers take action (Farmer et al. 2006). Yet despite the evident health needs of incarcerated people, there is no guarantee that providing care to incarcerated people is in itself a form of pragmatic solidarity. On the contrary, there is a long history of prison physicians, nurses and other clinicians perpetrating abuse and neglect (Chappell 2013). Nonetheless, there is also a history of health care workers ameliorating some harms of these institutions and helping keep incarcerated people alive in settings that cause defeat and illness.

The differences between the setting Farmer describes and what is revealed by our ethnographic work further demonstrate this complexity. In Farmer’s case, his team was invited and supported in working on a specific problem. In contrast, despite the rhetoric of the “Mission Possible” campaign, jail leadership subordinated health goals to the institutional prerogatives of security and order. As a result, some clinicians had little choice but to be complicit in providing sub-standard care and in practices of institutional brutality. Others, like Dr. Allen, willfully provided a bare minimum.

The context of the jail expansion plan also complicates Farmer’s proposition. Farmer states that the spirit of the service provision is critical: “Service delivery can be just that—or it can be pragmatic solidarity, linked to the broader goals of equality and justice for the poor” (Farmer 2010:448). In LA, however, service provision was linked instead to the goals of increasing the legitimacy of the LASD and building a new facility. This coupling is not specific to LA. In 2020, California’s legislature put forth a bill that would have directed state mental health funding to jails instead. Jail advocates in these cases have drawn on the rhetoric of benevolence and care in their requests for increased funds. James Kilgore (2014) and Ruth Gilmore refer to this strategy as “carceral humanitarianism” (Gilmore 2017), an organized response to the failings of jails that seeks to re-define their purpose and re-establish their legitimacy. Their analysis resonates with anthropologists of humanitarianism, who have demonstrated how humanitarian aid is typically framed as an apolitical moral imperative despite its complex political entanglements (Fassin 2011; Ticktin 2014). In the case of jails, jailers muster moral outrage on behalf of the mentally ill to justify expansion. As Orange County Sheriff said in 2019 in support of legislation to increase funding: “If we’re going to be a mental health hospital, we’re going to be a good one” (Pho 2019).

That carceral health care may be put to political ends other than justice highlights some limitations to Farmer’s analysis of prisons. In arguing for humane prison medicine, Farmer writes, “the state…has always arrogated the power to punish” (Farmer 2010:217). This transhistorical approach to the state—that understands its function and capacity for punishment as unchanging and inevitable—leaves us ill-equipped to understand how and why the U.S. built the largest system of incarceration in the world. Given these limitations, we need a better framework for “the state” to guide what pragmatic solidarity (Farmer 2010:441) might mean in U.S. jails and prisons.

## Organized Violence vs. Organized Care

Understanding the historical transformation that led to the U.S. carceral state’s expansion has been a central task of the field of critical prison studies. Geographer Ruth Gilmore’s *Golden Gulag*, a study of California with far-reaching implications, has become one of the foundations for this scholarship. Gilmore dispels the presumed linear relationship between crime and punishment, and likewise the apparent marginality of prisons to social life. The turn to prisons, she shows, is connected to changes in the global political economy and the changing meanings of “the state” in the U.S. Joining other critical scholars (Abrams 1988; Gupta 2012), she and Craig Gilmore argue against reifying the state. Rather, they define the state as the particular “ideological and institutional capacities” that “develop and change over time” through conflict and struggle (Gilmore and Gilmore 2008:143).

Thinking about the state this way focuses attention on how its ideological role and institutional capacities are not inevitable but *change* over time. For Gilmore and Gilmore, what changed between the 1970s and 1990s is that the U.S. state came to be increasingly defined ideologically by its ability to provide safety (for some) through punishment, while its previous role in promoting social welfare was transferred to the private sector. We see this history in action in LA: the county has had the financial capacity and, until recently, political support to build jails—but not the same capacity to build an alternate system of care.

Gilmore and Gilmore refer to the state’s increased institutional capacities for violence and incarceration as organized violence (Gilmore and Gilmore 2016). Here, they join incarcerated and formerly incarcerated intellectuals (Davis 1971, 2003; Jackson 1990 and others, as collected in James 2003) and other critical theorists (Rodriguez 2007) in connecting the transformation of the state to the US’ longer history of racial and class warfare. While police and prisons today appear normal and legitimate, scholar Naomi Murakawa, joining Gilmore and Gilmore, traces the origins of these institutions in extra-governmental, vigilante racial violence (Murakawa 2014). She argues that the promise of “protection” by the state from vigilante violence, such as lynchings, facilitated the rise of the organized violence (Gilmore and Gilmore 2016) of governmental agencies, which act with relative impunity.

This framework changes the object of struggle from just the excessive violence of carceral institutions—the types of excesses in which Farmer sought to intervene—to the *presence, size and reach* of institutions of organized violence (Gilmore and Gilmore 2016) as well. In LA, this framework broadens the lens to include police killings, jail deaths *and* the very fact of LA having the largest jail system in the world. In other words, it draws attention to how jails sometimes kill directly but also how they, indirectly, produce premature suffering and death at a population level via the broader “carceral mesh” of policing and incarceration (Wacquant 2010:82). Such a framework, additionally, foregrounds the hegemonic processes, both political and ideological, that have helped make these carceral institutions appear normal.

The pivot to thinking of police and prisons as the organized violence of the state (Gilmore and Gilmore 2016) has direct implications for strategic and extra-clinical action. First, institutions of violence cannot be straightforwardly reformed. And second, if the state has now come to be defined by its organized violence (Gilmore and Gilmore 2016), its ideological function and institutional capacities could also then be put, instead, to other, non-violent ends, such as providing public goods and services. This framework has become foundational for movements organizing for the abolition of police, jails and prisons.

We can use this framework for thinking about how the state is contested and re-imagined in JusticeLA Coalition’s campaign for “care, not cages.” On one hand, they highlighted the disproportionate size LASD had claimed of the public budget and how the repeated institutional abuses by LASD deputies revealed structural, rather than individual, problems with the department that could not be solved through reform. So, they sought to diminish the power of the LASD, an institution of organized violence. On the other, the coalition also engaged in a positive project of seeking to build alternate systems and institutions of care (i.e. *care*, not cages).

As anthropologists and feminist scholars have pointed out, care is an elusive and contested concept, alternatively describing a form of control and violence and of interdependence and solidarity. In the past decade, organizers for abolition have foregrounded “care” as a central part of its practical strategy and affirmative vision for transformation. They have drawn on care’s Black feminist and activist genealogies, synthesized in Saidiya Hartman’s influential assertion that “care is the antidote to violence” (Hartman 2017). Following Hartman, Mariame Kaba has described care as “a relationship practice” that can “connect people to participate in projects of freedom,” via defense campaigns and other tactics of support for criminalized and incarcerated people (Kaba 2021).

In LA, abolitionist organizers envisioned and fought for a model of “care” resonant with Hartman and Kaba’s theorizing. Ideologically, the care JusticeLA sought was in principled opposition to the organized violence of the police and jails (Gilmore and Gilmore 2016). Against efforts to fold care into the everyday functioning of carceral institutions, they emphasized the tension between care and policing and jails and thus the necessity of uncoupling care from spaces predefined by state violence and instead building a system of care that would not be predicated on police contact or arrest. This ideological insistence led the County to pivot away from their plan for a more “caring” jail system and towards a “care first, jail last” approach. The County decreased support for the LASD and drew attention to the responsibility of other county agencies. For example, it helped prompt the Supervisors to study the County’s absence of public psychiatric services, develop a fund inaccessible to law enforcement agencies and strengthen its Office of Diversion and Re-Entry (ODR).

In tandem with their efforts to redefine the County’s meaning of care, JusticeLA sought to build the state’s *capacity* to provide this alternate form of care. They participated in the County’s Alternatives to Incarceration Work Group and helped co-author its final report, a blueprint for programs that the County could pursue instead of jails. The programs outlined in this report included those in which the jail health service had an important role, such as expanding substance use disorder treatment programs (including MAT and adequate withdrawal management) and improving re-entry services. So, the report did not oppose improving the jail health system; rather, it sought to strengthen the provision of jail health care but within a broader strategy of de-carceration and improving public health.

In our ethnographic work, we saw first-hand the dynamism of the “state”—between what the state was doing and what else it could be called upon to do—and how health workers could play a role, beyond the clinic, in changing the definitions of the state’s function and capacity. Whether the jail should provide MAT services or whether people with SMIs should receive services from institutions other than jails and prisons were sites of active contestation. Similarly, we observed how some health experts, such as the hired health consultants, offered support for increasing the jail’s capacity for care—but others insisted the state should increase its capacity for community care. Due to the success of the JusticeLA campaign, the Board of Supervisors increased the capacity of care for criminalized and incarcerated people in ways clinicians had been unable to achieve within the jail. Public campaigning also directly led to increases to the ODR budget, which diverts people with serious mental illness into supportive housing and programming. JusticeLA’s broader struggle for “care” had thus changed the role and function of the jail and the jail health service therein.

We suggest that these broader goals of JusticeLA and other community organizations may be descriptively and analytically understood as seeking to transform the ideological and institutional capacities of the state—away from organized violence (Gilmore and Gilmore 2016) and towards an alternate vision that we call “organized care.” If the LA county public sector had come to be defined by its institutions of organized violence (Gilmore and Gilmore 2016), like jails, JusticeLA asked that it be redefined according to its ability to promote health and well-being through alternative, non-violent institutions. Our ethnographic work demonstrated how jails, and the ideologies of (un)deservingness and relations of force that accompanied them, decreased the capacity for and demobilized care. The organizing by JusticeLA, in contrast, reclaimed the meaning of health and care and built capacity within and without jails. They asserted that criminalized and incarcerated people, and their communities, deserved more from the state than further policing and incarceration. They demanded a state with the institutional capacities to provide forms of care ideologically and practically opposed to the organized violence (Gilmore and Gilmore 2016) of policing and incarceration. Organized care, in short, is one way to understand the organizers’ affirmative vision of what the state could provide in place of institutions of organized violence (Gilmore and Gilmore 2016).

Putting jail clinical work into this broader context of political struggle—between efforts to strengthen institutions of organized violence (Gilmore and Gilmore 2016) and those seeking to build institutions of organized care in their place—helps resolve the tensions we experienced in trying to conceptualize how health workers could offer pragmatic solidarity (Farmer 2010:441) to incarcerated people. The goals of equality and justice at the forefront of the movement for “care, not cages” are no different than the goals typical of projects advocated by Farmer. What organized care brings, however, is a framework to recognize how pragmatic solidarity (Farmer 2010:441) is most meaningful when it does not take carceral institutions, or any other institutional structure, in which the clinic is embedded, as inevitable. Instead, health workers can engage in a “liberatory solidarity” (Dubal, Samra, and Janeway 2021) that connects the frustrations they experience inside the clinic to political struggles already taking place outside the clinic. With this broader vision, clinicians can take a position in transformations already unfolding.

## Conclusion

In this paper, we demonstrated the potential for engaged clinical ethnography to contribute to struggles for social change. We show in our ethnographic vignettes the challenges posed by carceral institutions to acts of pragmatic solidarity (Farmer 2010:441), within and without the clinical encounter. The LASD and the jail health service worked together to limit the capacity of health care and subordinate the meaning of care to institutional prerogatives. The LASD and County Supervisors also put care in the service of the reproduction of a status quo of mass incarceration. From such a position, the best-intentioned clinicians may righteously “tweak Armageddon” (Gilmore 2007:48)—but achieve not much beyond that.

Such a predicament led us to bring together Farmer’s concept of pragmatic solidarity (Farmer 2010:441) with Gilmore and Gilmore’s framework of organized violence (Gilmore and Gilmore 2016) and the social movements struggling for “organized care.” Synthesizing these two frameworks not only aligns with Farmer’s original formulation but also broadens the call for “care, not cages.” The organizers’ demand, we suggest, is equally a call for the dismantling of carceral institutions and for the building of life-affirming institutions for criminalized and incarcerated people. In such a project, health workers may have a crucial role to play.

An important caveat to our study is that U.S. jails and jail health systems, while sharing core features, differ in significant ways. Though the LA jail system has national implications, it is not necessarily representative of all jails in the U.S. and certainly not the world, particularly with respect to mental health. Not all U.S. jails are operated by Sheriff Departments and not all jail health systems are co-managed with county health systems. Moreover, this study described a setting in which a social movement around the object of study already existed. And while this paper engaged the broad vision of this movement, it did not elaborate its inner tensions and conflicts. Finally, this study did not include the contributions of incarcerated people who engage in their own care work (Burton 2021).

We hope, nonetheless, that our engaged clinical ethnography models how medicine and anthropology may collaborate towards goals that reach beyond their own disciplinary boundaries and beyond the clinical setting. Ethnography, in our case, did not offer a fix to the constraints and limits on providing care in an ethically compromised setting through its own thickness of description and analysis. Rather, our engaged clinical ethnography drew us out of the clinical encounter and into wider fields of action and struggle. Embedding ourselves within and informed by these social struggles, we oriented our clinical ethnography to assisting these movements. We sought to demonstrate how clinicians and ethnographers may not only wade into politically fraught and unstable ground, but also join others seeking to “shake the ground” altogether (Gilmore 2007:248).

## Footnotes

1 For foundational prison ethnographies in the U.S., see, for example, Clemmer (1940), Sykes (1958) and Olin Wright (1973). For a study focused on jails, see Irwin (1985).

2 Medication-Assisted Treatment, or “MAT,” describes medication-based treatments for substance use disorders, such as methadone or buprenorphine for opioid use disorders.

3 The extent to which opioid withdrawal is life-threatening is not straightforward. While there is less risk of dying than during alcohol withdrawal, the risk is not zero, due to associated comorbidities; it is also known to be excruciatingly painful (Bourgois and Schonberg 2009).

4 See McCann (2022) and Roth (2018), as examples, which draw on Torrey et al. (2010). In 2019, there was an average of 5676 inmates receiving mental health treatment (LASD 2019); the largest state psychiatric hospital in California has a capacity of 1527 beds (California Department of State Hospitals 2023).

5 Meanwhile, the Chief Medical Officer of the LA jail system was hired to work as an expert witness by the U.S. private prison company GEO Group in a class action lawsuit against an immigration detention facility the company managed. His expertise was established by his competence responding to Covid-19 in the LA jails. GEO Group was ruled to be untrustworthy and required to release many detainees (Castillo 2022).
